# Quantifying propagation of DNA methylation and hydroxymethylation with iDEMS

**DOI:** 10.1038/s41556-022-01048-x

**Published:** 2023-01-12

**Authors:** Kathleen R. Stewart-Morgan, Cristina E. Requena, Valentin Flury, Qian Du, Zoe Heckhausen, Petra Hajkova, Anja Groth

**Affiliations:** 1 Novo Nordisk Foundation Center for Protein Research (CPR), Faculty of Health and Medical Sciences, University of Copenhagen, Copenhagen, Denmark; 2 Biotech Research and Innovation Centre (BRIC), Faculty of Health and Medical Sciences, University of Copenhagen, Copenhagen, Denmark; 3 MRC London Institute of Medical Sciences (LMS), London, UK; 4 Institute of Clinical Sciences (ICS), Faculty of Medicine, Imperial College London, London, UK; 5 Garvan Institute of Medical Research, Sydney, New South Wales Australia; 6 St Vincent’s Clinical School, University of New South Wales, Sydney, New South Wales Australia

**Keywords:** DNA methylation, Chromatin, Embryonic stem cells, DNA replication, Post-translational modifications

## Abstract

DNA methylation is a critical epigenetic mark in mammalian cells. Many aspects of DNA methylation maintenance have been characterized; however, the exact kinetics of post-replicative methylation maintenance remain a subject of debate. Here we develop isolation of DNA by 5-ethynyl-deoxyuridine labelling for mass spectrometry (iDEMS), a highly sensitive, quantitative mass spectrometry-based method for measuring DNA modifications on metabolically labelled DNA. iDEMS reveals an unexpectedly hemi-methylated landscape on nascent DNA. Combining iDEMS with metabolic labelling reveals that methylation maintenance is outpaced by cell division in mouse embryonic stem cells. Our approach shows that hydroxymethylation is perpetually asymmetric between sister strands in favour of the parental, template strand. iDEMS can be coupled with immunoprecipitation of chromatin proteins, revealing features of DNA methylation–histone modification crosstalk and suggesting a model for interplay between methylation and nucleosome assembly. iDEMS therefore elucidates long-standing questions about DNA modification propagation and provides an important orthogonal technology to understanding this process in dynamic cellular contexts.

## Abstract (web summary)

Stewart-Morgan et al. present isolation of DNA by 5-ethynyl-deoxyuridine labelling for mass spectrometry, a highly sensitive, quantitative mass spectrometry-based method for measuring DNA modifications on metabolically labelled DNA. They apply it to study DNA methylation and hydroxymethylation propagation.

## Main

DNA methylation (5-methyl-deoxycytidine, 5mdC) is a key repressive epigenetic modification in mammals and other eukaryotes. Through altering DNA accessibility to transcriptional machinery, DNA methylation modulates gene expression within the cell^1^. Besides its role in transcriptional regulation, DNA methylation participates in well-established crosstalk with other aspects of the epigenetic landscape, including histone modifications. In euchromatin, DNA methylation is found in active gene bodies, where it helps prevent spurious transcription initiation along with histone H3 lysine 36 trimethylation (H3K36me3) (ref. ^2^); in heterochromatin it coincides with H3K9 di- and trimethylation, and helps to compact chromatin to maintain repression and genome stability^3^. DNA methylation can be oxidized to hydroxymethylation (5hmdC), which plays crucial roles at regulatory elements^4^. Given their roles in epigenetic regulation, propagating both the methylome and the hydroxymethylome between cell divisions is key to epigenetic cell memory. Loss of DNA methylation causes genome instability that can result in aneuploidy, chromosomal translocation and cell death. Notably, misregulation of DNA methylation is pervasive in cancer^4,5^ and ageing^6^.

DNA methylation is typically symmetric between complementary DNA strands, decorating palindromic CpG dinucleotides^7^. DNA synthesis during S phase creates hemi-methylated DNA, on which methylation must be re-established to restore symmetric methylation and thus propagate the cell methylome faithfully to daughter cells. DNA methylation maintenance is classically described as the mechanisms that ‘copy’ the methylation pattern from the parental strand onto the newly synthesized DNA strand. This process is dominated by the activity of the maintenance DNA methyltransferase DNMT1 and its cofactor UHRF1 (ref. ^7^). However, it is now widely accepted that DNMT3A and DNMT3B, traditionally considered de novo methyltransferases, also play a role in methylation maintenance, especially at repetitive elements^8,9^. While DNA methylation maintenance is well studied, little remains known about propagation of DNA hydroxymethylation, which was first characterized as part of the active DNA demethylation pathway in mammals^10^. Apart from its role as an intermediate in oxidative DNA demethylation, hydroxymethylation is also considered to act as a stable epigenetic mark in many genomic and cellular contexts^11–13^. In contrast to methylation, hydroxymethylation is often asymmetric between DNA strands^14^, meaning a molecular maintenance mechanism analogous to hemi-methylation recognition is unlikely to underpin its propagation across cell divisions.

DNA methylation maintenance has been proposed to proceed in two phases: a replication fork-coupled phase followed by a replication fork-uncoupled phase^15^. The replication fork-coupled phase entails direct recruitment of the DNA methyltransferase machinery to the replication fork. To this end, DNMT1 interacts directly with PCNA via a PCNA-binding motif^16^, and its cofactor UHRF1 binds LIG1 (ref. ^17^). However, loss of PCNA binding by DNMT1 causes only mild hypomethylation^18^, indicating that this recruitment mechanism is not an absolute requirement for methylation maintenance. Similarly, ablating UHRF1’s interaction with LIG1 does not cause complete loss of methylation^17^, although UHRF1 is critically required for methylation^19^. A study combining genetic complementation and measurements of methylation restoration kinetics by next-generation sequencing found that slowed methylation rates caused by loss of replication fork-coupled maintenance are counteracted by the subsequent replication fork-uncoupled phase^15^, indicating there exist multiple and compensatory pathways to maintain methylation.

DNA methylation maintenance occurs not on naked DNA, but in the context of chromatin^7^. In addition to direct interaction with the replisome, DNA methyltransferase complexes are recruited to DNA by the presence of specific chromatin features. UHRF1 binds hemi-methylated DNA sites^19,20^ and ubiquitylates histone H3 at K14, K18 and/or K23 (ref. ^21^). This ubiquitylation is specifically recognized by DNMT1 and thus represents a means to direct maintenance methylation only to hemi-methylated sites^21,22^. Early- and late-replicating DNA is embedded within very different chromatin environments, including differing methylation levels^23,24^, and different genomic regions probably exploit distinct methyltransferase recruitment mechanisms. DNMT1 binds to the heterochromatin factor HP1 (ref. ^25^), and UHRF1 binds to H3K9me2/3 (refs. ^26,27^), both of which are enriched in late-replicating DNA. It may be that late-replicating regions rely on additional mechanisms of methyltransferase recruitment because DNA methylation is more functionally important in these regions, or that such mechanisms counterbalance the proportionally smaller window of time in which methylation restoration can occur before mitosis.

Multiple studies have observed that DNA methylation is progressively lost over multiple cell divisions, especially from late-replicating DNA^23,28^. This suggests that DNA methylation maintenance kinetics are slow compared with cell cycle progression, with repeated failure to restore the methylome before the following S phase resulting in eventual loss of methylation. Recently, several studies have developed methods to assess DNA methylation restoration on replicated DNA through sequencing approaches^29^. Two studies in human embryonic stem cells gave conflicting results, with one study reporting restoration of the methylome within 20 min of replication^30^ and the other showing slower kinetics, with restoration of bulk levels seen only 4 h post-replication^31^. A third study using HeLa cells saw full restoration of methylation within 10 h of replication, but reported that more than 80% of the methylome was restored within 30 min (ref. ^15^). These reports have provided important insights into how multiple chromatin factors contribute to methylation maintenance. However, these differing results have left the kinetics of methylation maintenance unclear. Additionally, none of these approaches could inform on the kinetics of hydroxymethylation post-replication.

In this Technical Report, to resolve DNA methylation maintenance kinetics and directly quantitate DNA modification abundance on replicated DNA, we developed isolation of DNA by 5-ethynyl-deoxyuridine labelling for mass spectrometry (iDEMS). iDEMS resolves post-replicative methylation kinetics on both the parental and the newly replicated strands, showing that restoration is slow. iDEMS in synchronized cells and whole-genome sequencing of sorted G2/M and G1 populations both show that methylation restoration is incomplete at mitosis. We additionally profile post-replicative hydroxymethylation kinetics directly on replicated DNA. We combine our strand-separation method with metabolic labelling to reveal that methylation restoration entails post-replicative methyl deposition on both the newly replicated and the parental strand. Finally, by applying iDEMS on immunoprecipitated DNA, we show that methylation levels in nascent chromatin differ on the basis of chromatin context, with H3K9me3-associated DNA showing higher methylation immediately after replication. iDEMS is therefore a valuable and versatile method that provides important insights into how DNA modifications are propagated across cell divisions.

## Results

### iDEMS measures DNA modifications on labelled DNA

**Fig. 1:**
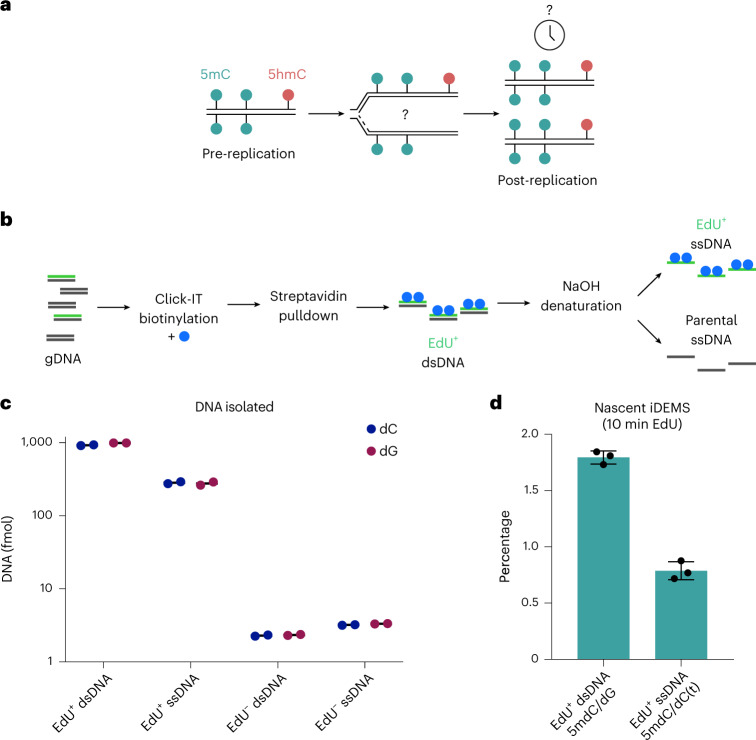
iDEMS isolates replicated DNA with high purity. **a**, The kinetics of post-replicative restoration of DNA methylation and hydroxymethylation are unclear. **b**, Scheme of iDEMS method. **c**, DNA abundance in EdU^+^ dsDNA, EdU^+^ ssDNA, EdU^−^ dsDNA and EdU^−^ ssDNA samples. Points represent technical replicates. **d**, Methylation levels measured in nascent (10 min pulse) iDEMS, in EdU^+^ dsDNA and EdU^+^ ssDNA. In **d**, data are presented as mean ± s.d. from three biological replicates. Numerical source data are provided. Source data

**Extended Data Fig. 1:**
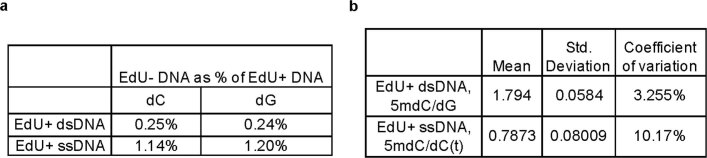
Extended Data Related to Fig. 1. **a**) Calculations of potential contamination in iDEMS samples, as assessed by dividing the EdU- controls in Fig. 1c from their matched EdU+ samples. **b**) Means, standard deviations, and coefficients of variation for data in Fig. 1d. Numerical source data are provided. Source data

Although DNA methylation has been extensively characterized, the kinetics of its deposition on daughter strands after DNA replication remains unclear (Fig. 1a). The post-replication dynamics of its oxidized derivative, hydroxymethylation, are even less understood (Fig. 1a). To directly and quantitatively measure DNA modifications after replication, we developed iDEMS, a strategy to purify DNA labelled during replication for analysis by liquid chromatography linked to tandem mass spectrometry (LC–MS/MS). Using Click-IT technology followed by streptavidin pulldown, we purified 5-ethynyl-deoxyuridine (EdU)-labelled double-stranded DNA (EdU^+^ dsDNA). Further separation of EdU^+^ dsDNA samples into the newly synthesized (EdU^+^ single-stranded DNA (ssDNA)) and parental ssDNA strands allowed us to measure the kinetics of post-replicative methylation and hydroxymethylation restoration quantitatively and in high resolution (Fig. 1b). By using stringent purification conditions, iDEMS achieved approximately 99% purity, as assessed by comparison with a negative control using unlabelled (EdU^−^) genomic DNA (gDNA) as input (Fig. 1c and Extended Data Fig. 1a). This method was robust, with EdU pulses as short as 10 min in mouse embryonic stem cells (mESCs) (Fig. 1d and Extended Data Fig. 1b).

### Major role of fork-uncoupled maintenance post-replication

**Fig. 2:**
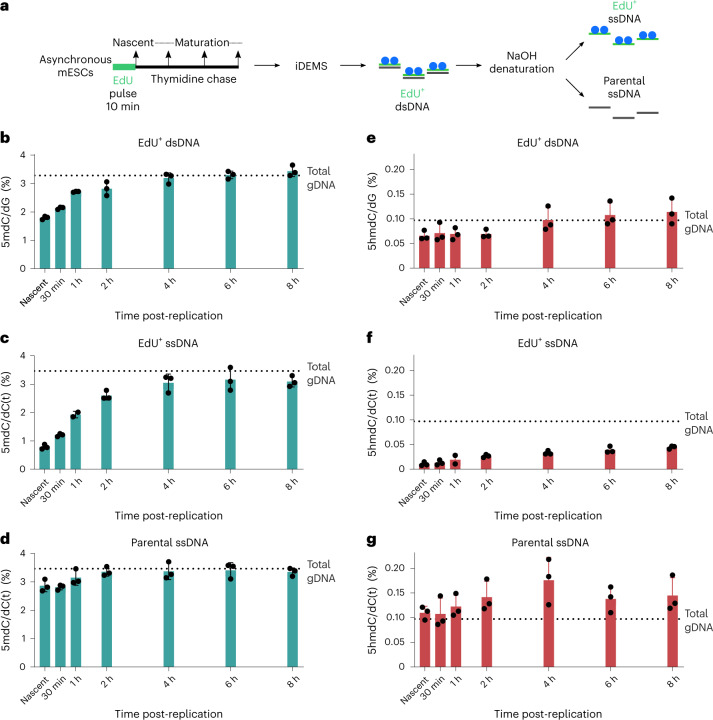
iDEMS reveals methylation and hydroxymethylation maintenance kinetics post-replication. **a**, Scheme of mESC timecourse. **b**, Bar chart of methylation in EdU^+^ dsDNA. **c**, Bar chart of methylation in EdU^+^ ssDNA. **d**, Bar chart of methylation in parental ssDNA. **e**, Bar chart of hydroxymethylation in EdU^+^ dsDNA. **f**, Bar chart of hydroxymethylation in EdU^+^ ssDNA. **g**, Bar chart of hydroxymethylation in parental ssDNA. In **b**–**g**, data are presented as mean ± s.d. from three biological replicates. In **b** and **e**, levels are calculated as percentage of dG. In **c**, **d**, **f** and **g**, levels calculated as percentage of dC(t). Numerical source data are provided. Source data

**Extended Data Fig. 2:**
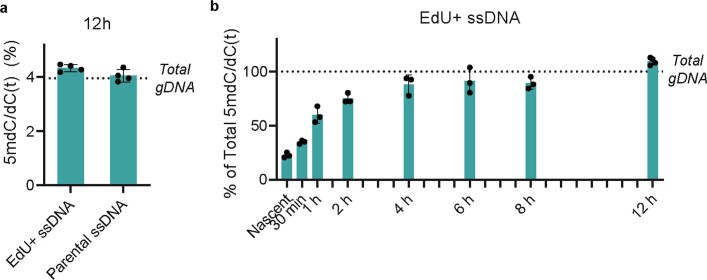
Extended Data Related to Fig. 2. **a**) Barchart of methylation in EdU+ ssDNA and parental ssDNA at 12 h post-replication. **b**) Barchart of methylation in EdU+ ssDNA, calculated as a percentage of matched total gDNA. Data are presented as mean values + /− SD from three or four biological replicates as indicated. Numerical source data are provided. Source data

To analyse newly replicated DNA, we collected samples immediately following a pulse of the thymidine analogue EdU; to track re-methylation kinetics after replication, we chased this pulse for up to 8 h (Fig. 2a). As the doubling time of our mESCs is 12 h, this timecourse represents two-thirds the total length of the mESC cell cycle, tracking methylation and hydroxymethylation beyond replication and into the following cell cycle. Methylation on replicated, EdU^+^ dsDNA reached gDNA levels within 4 h of replication (Fig. 2b). This observation is broadly in agreement with previous work showing a marked lag between replication and complete methylation restoration^15,31^. Kinetics on the EdU^+^ ssDNA strand mirrored that seen on EdU^+^ dsDNA, with methylation gains levelling off after 4 h (Fig. 2c). To complement our timecourse, we additionally performed iDEMS 12 h after replication, representing one full cell cycle in mESCs. At 12 h, methylation levels on EdU^+^ ssDNA matched or exceeded those seen in both parental ssDNA and total gDNA (Extended Data Fig. 2a,b), indicating that a small amount of methylation is additionally gained between 8 h and 12 h post-replication on the newly synthesized DNA strand. Therefore, while population methylation levels are reached on dsDNA within 4 h of replication, the newly replicated DNA strand does not accomplish this until one full cell cycle later.

Notably, within the EdU^+^ ssDNA samples, the nascent timepoint contained 25–30% of the methylation seen at 8 h (Extended Data Fig. 2b). The EdU^+^ ssDNA in our nascent timepoint is a maximum of 10 min old (the interval of our EdU pulse), and therefore estimated to be 30 kb or less from an active replication fork on the basis of the rate of DNA synthesis^32^. This indicates that replication fork-uncoupled mechanisms play a more prominent role in methylation maintenance than previously suggested^15,30^. Intriguingly, parental ssDNA methylation levels were not constant, instead progressively gaining methylation post-replication (Fig. 2d). Additionally, parental ssDNA from the nascent and 30 min timepoints had statistically less methylation than the gDNA average (Supplementary Table 1). This implies that de novo methylation of the parental strand additionally contributes to maintaining methylation levels on post-replicative DNA.

Our iDEMS approach simultaneously analysed methylation and hydroxymethylation on EdU-labelled DNA. We found that overall restoration kinetics between methylation and hydroxymethylation were similar, and that genomic hydroxymethylation levels were achieved on EdU^+^ dsDNA within 4 h of replication (Fig. 2e). However, hydroxymethylation profiles on EdU^+^ ssDNA and parental ssDNA showed that the mode underlying restoration of these two modifications clearly differed. Hydroxymethylation on the newly replicated strand remained low throughout the timecourse, equalling less than half of population levels 8 h after replication (Fig. 2f). In contrast, hydroxymethylation on the parental strand reproducibly exceeded the genome average within 1 h of replication (Fig. 2g). This shows that oxidation of the parental strand contributes substantially to overall hydroxymethylation levels, in contrast with methylation, whose increase in post-replicative DNA is mainly due to modification of the newly synthesized strand.

### Alterations in methylation metabolism across S phase

**Fig. 3:**
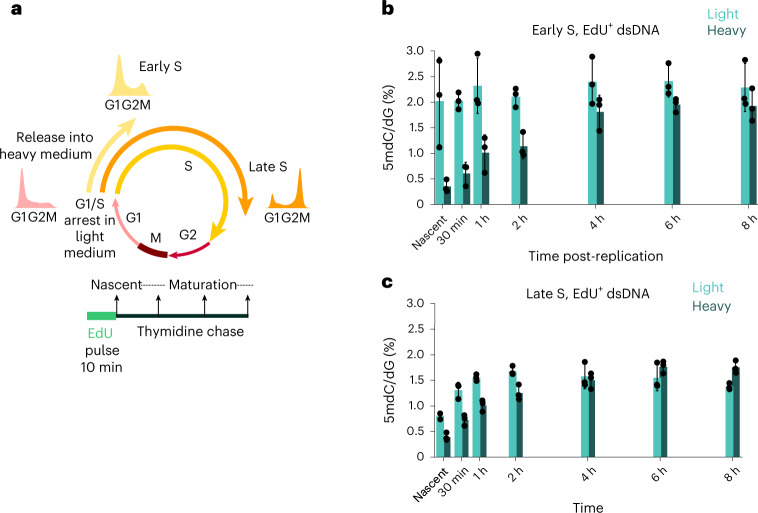
SILAC-iDEMS reveals differential methylation turnover in early- and late-replicating DNA. **a**, Scheme of synchronization and labelling strategy for the early and late S timecourses. **b**, Bar chart of heavy and light methylation in early-replicating DNA. **c**, Bar chart of heavy and light methylation in late-replicating DNA. Data are presented as mean ± s.d. from three biological replicates. Numerical source data are provided. Source data

An advantage of a mass spectrometry approach to methylation maintenance is that bona fide new methylation deposition can be tracked through metabolic labelling. As methionine is the metabolic source of DNA methylation and its oxidized derivatives, changing standard media for media containing only heavy isotope-labelled methionine before EdU labelling constitutes an orthogonal method for tracking methylation kinetics after DNA replication. We incorporated this labelling approach with cell synchronization at the G1/S boundary and release to track methylation restoration in early- and late-replicating regions (Fig. 3a). Notably, the cell synchronization method barred analysis of hydroxymethylation in these data, since synchronization has been shown to alter hydroxymethylation levels^13^.

**Extended Data Fig. 3:**
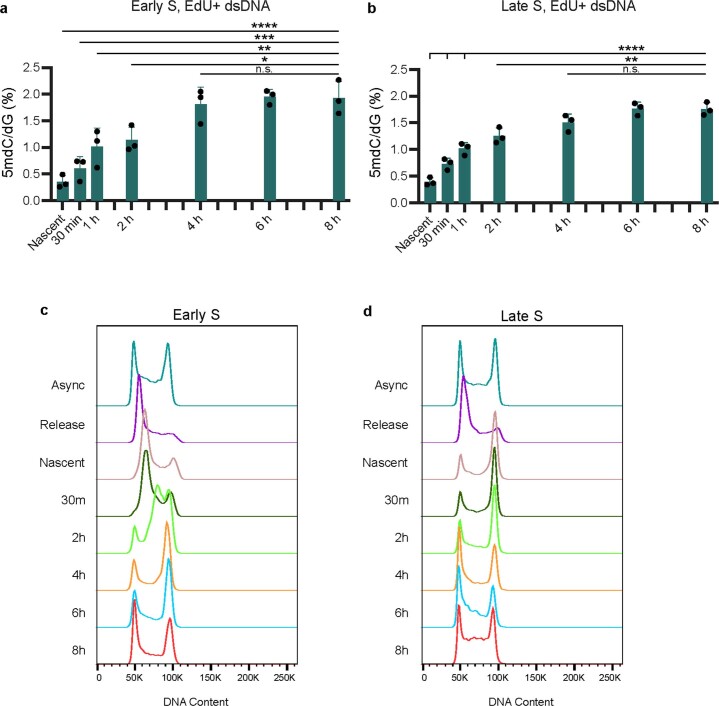
Extended Data Related to Fig. 3. **a**) Barchart of heavy methylation in early replicating DNA. **b**) Barchart of heavy methylation in late-replicating DNA. **c**) FACS plots showing DNA content from the total cell population during the early S timecourse. **d**) FACS plots showing DNA content from the total cell population during the late-S timecourse. In a-b), data are presented as mean values + /− SD from three biological replicates. P-values calculated using one-way ANOVA, adjusting for multiple comparisons: *, p < 0.05; **, p < 0.01; ***; p < 0.001 ****, p < 0.0001. Exact adjusted p-values for a): 8 h v Nascent: p < 0.0001; 8 h v 30 min: p = 0.0001; 8 h v 1 h: p = 0.0036; 8 h v 2 h: p = 0.0109; 8 h v 4 h: p = 0.9849; 8 h v 6 h: p = 0.9998. Exact adjusted p-values for b): 8 h v Nascent: p < 0.0001; 8 h v 30 min: p < 0.0001; 8 h v 1 h: p < 0.0001; 8 h v 2 h: p = 0.001; 8 h v 4 h: p = 0.1099; 8 h v 6 h: p = 0.9999. Numerical source data are provided. Source data

As measured by heavy isotope methylation incorporation, the rate of methylation in early- and late-replicating regions approximately mirrored that measured genome wide, with most gains seen between nascent and 4 h (Fig. 3b,c and Extended Data Fig. 3a,b). Therefore, the rate of re-methylation is largely uniform genome wide, but generally slow. This suggests that, while early-replicating regions restore methylation before mitosis, a proportion of late-replicating regions probably do not restore their methylation levels until the following cell cycle (Extended Data Fig. 3c,d). The kinetics of this re-methylation seemed to differ slightly, with early-replicating DNA showing a big gain in heavy methylation between 2 h and 4 h, while late-replicating DNA showed a more constant increase. Intriguingly, while light methylation remained constant in early-replicating regions, as expected, late-replicating regions saw gains in both light and heavy methylation early after replication, up to the 2 h timepoint (Fig. 3c). This gain in light methylation suggests that, in late S phase, methionine within cells may be depleted, necessitating salvage pathways to recycle methionine donors and supply the methyltransferase machinery in the early stages of post-replicative maintenance.

### DNA methylation maintenance is incomplete at mitosis

**Fig. 4:**
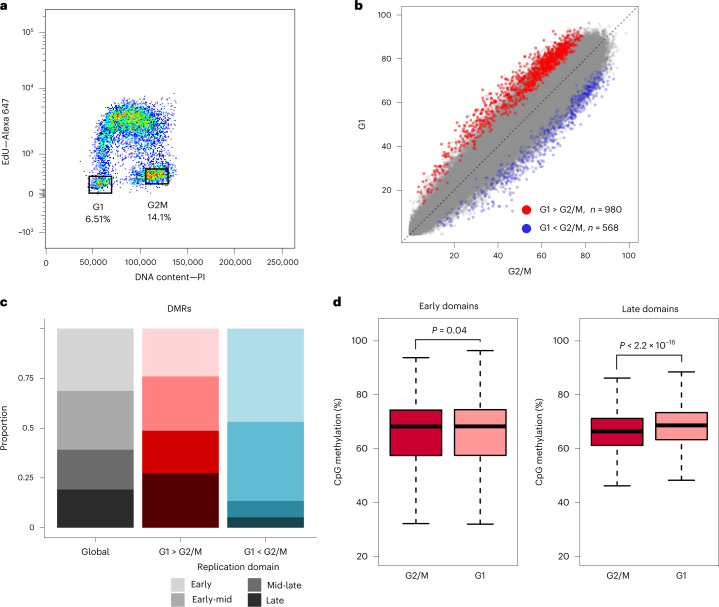
DNA methylation levels are not restored by mitosis. **a**, Sorting strategy to isolate pure populations of mESCs in G1 and G2/M. Numbers represent the percentage of cells in each gate. PI, propidium iodide. **b**, Percentage of CpG methylation genome wide in G2/M versus G1 cells. Red dots: G1 > G2/M DMRs; blue dots: G1 < G2/M DMRs. **c**, Stacked bar chart showing proportion of replication domains overlapping quantifiable 100 CpG windows globally (left), in G1 > G2/M DMRs (centre) and in G1 < G2/M DMRs (right). **d**, CpG methylation levels in early (left) and late (right) replication domains in sorted G2/M and G1 populations. Black line: median; boxes: 25th–75th percentiles; dashed lines: 1.5× interquartile range. *P*-values are indicated in each plot. EM-seq data in **b**–**d** calculated using 100-CpG windows quantified in all datasets. DMRs identified using logistic regression, *P* < 0.05. *P* values calculated using two-sided Mann–Whitney *U* test. Replication timing data from ref. ^45^. Numerical source data are provided. Source data

**Extended Data Fig. 4:**
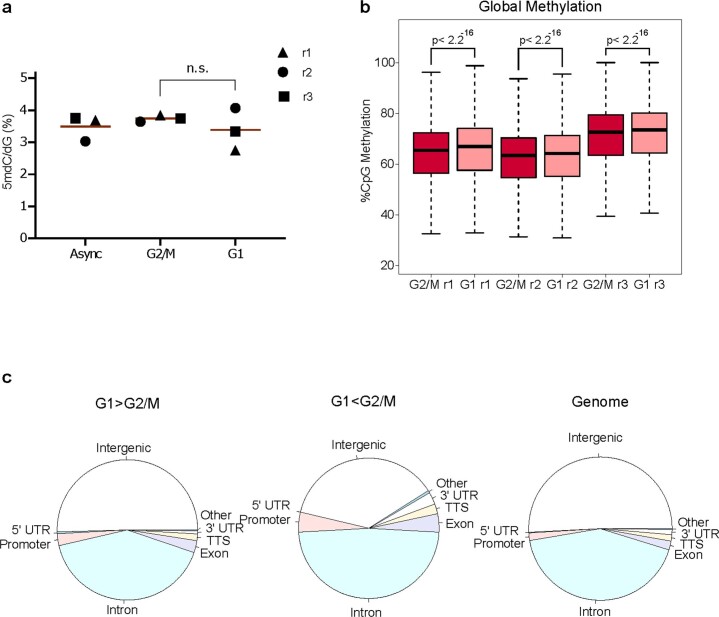
Extended Data Related to Fig. 4. **a**) LC-MS analysis of sorted G1 and G2/M mESCs, plus an asynchronous control. P-values calculated using one-way ANOVA. **b**) Boxplots of global methylation levels across G1 and G2/M replicates. Black line: median; boxes: 25^th^–75^th^ percentiles; dashed lines: 1.5x interquartile range. *P*-values are indicated in the plot. Calculated using 100-CpG windows quantified in all datasets. **c**) Genome annotations of G1>G2/M DMRs (left), G1<G2/M DMRs (center), and the genomic distribution of the features annotated, for reference (right). P-values calculated using two-sided Mann-Whitney test. Numerical source data are provided. Source data

Our cell-synchronization data indicated that methylation maintenance continues past mitosis, predicting that DNA methylations levels should be higher in G1 than in G2/M. To test this, we sorted pure populations of G1 and G2/M mESCs using flow cytometry (Fig. 4a). The iDEMS data from our early and late S timecourses predicted that only late-replicating regions would be incompletely methylated in the following G1 phase, and that the majority of the methylome would be restored by mitosis. Consistent with this, global analysis of DNA methylation in these populations by LC–MS/MS showed no significant differences between G1 and G2/M populations (Extended Data Fig. 4a). To probe this further, we generated genome-wide methylation profiles of G1 and G2/M populations using enzymatic methyl-seq (EM-seq)^33^. Within matched replicates, G1 EM-seq consistently showed slightly higher global methylation than G2/M (Extended Data Fig. 4b). We identified 1,548 differentially methylated regions (DMRs) between the cell cycle phases, with the majority being hypermethylated in G1 cells (Fig. 4b). G1-hypermethylated DMRs disproportionately overlapped with late-replication domains, while G2/M-hypermethylated DMRs were enriched in early-replication domains (Fig. 4c). Neither DMR set was notably enriched for particular genomic loci (Extended Data Fig. 4c) or gene ontologies. Targeted analysis of replication domains revealed that domains that replicate early showed no methylation differences between G1 and G2/M, but late-replicating domains had higher methylation in G1 cells (Fig. 4d). Therefore, post-replication, the mESC methylome is largely restored by mitosis, but fully restored only in daughter cells, with late-replicating domains being the last to return to symmetric DNA methylation on both DNA strands.

### Both DNA strands are continuously modified post-replication

**Fig. 5:**
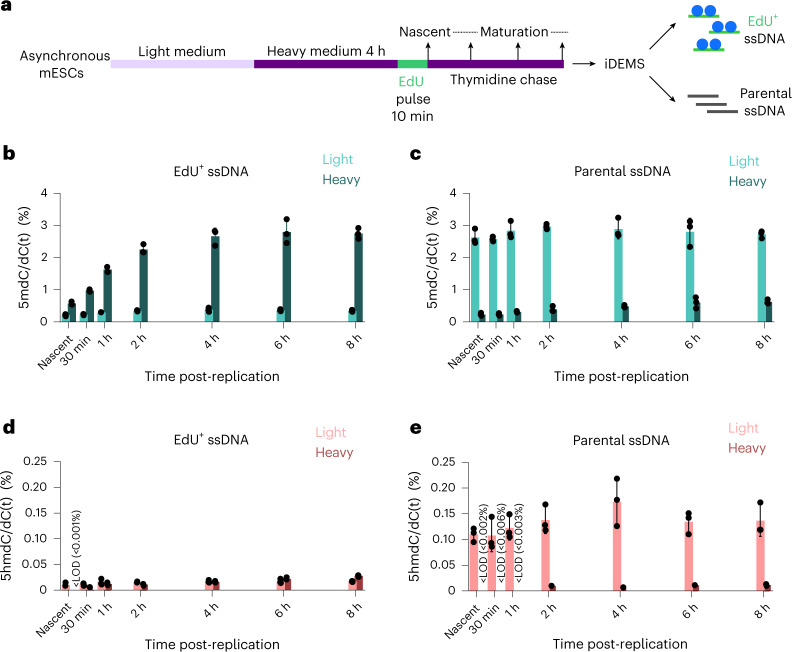
Both parental and newly replicated DNA strands accumulate new methylation and hydroxymethylation post-replication. **a**, Scheme of SILAC-iDEMS strategy in asynchronous mESCs. **b**, Bar chart of heavy and light methylation in EdU^+^ ssDNA. **c**, Bar chart of heavy and light methylation in parental ssDNA. **d**, Bar chart of heavy and light hydroxymethylation in EdU^+^ ssDNA. **e**, Bar chart of heavy and light hydroxymethylation in parental ssDNA. In **b**–**e**, data are presented as mean ± s.d. from three biological replicates. <LOD, below limit of detection. Numerical source data are provided. Source data

**Extended Data Fig. 5:**
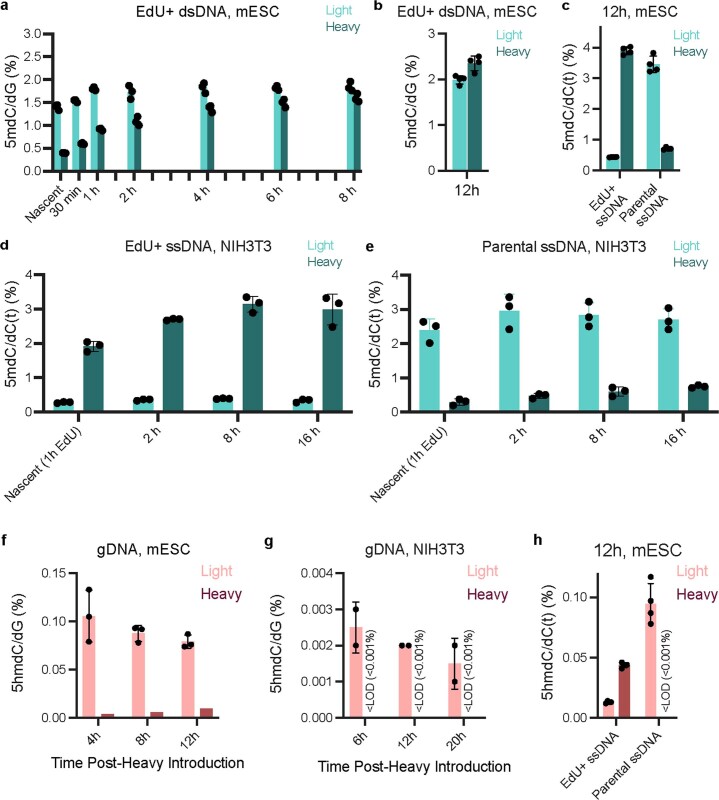
Extended Data Related to Fig. 5. **a**) Barchart of heavy and light methylation in mESC EdU+ dsDNA. **b**) Barchart of heavy and light methylation in EdU+ dsDNA at 12 h post-replication. **c**) Barchart of heavy and light methylation in EdU+ ssDNA and parental ssDNA at 12 h post-replication. **d**) Barchart of heavy and light methylation in NIH3T3 EdU+ ssDNA. **e**) Barchart of heavy and light methylation in NIH3T3 parental ssDNA. **f**) Barchart of heavy and light hydroxymethylation in mESC genomic DNA after heavy media switch for one cell cycle (12 h). **g**) Barchart of heavy and light hydroxymethylation in NIH3T3 genomic DNA after heavy media switch for one cell cycle (20 h). **h**) Barchart of heavy and light hydroxymethylation in EdU+ ssDNA and parental ssDNA at 12 h post-replication. In a-h), data are presented as mean values +/− SD from three or four biological replicates as indicated. <LOD, below limit of detection. Numerical source data are provided. Source data

Applying our metabolic labelling and strand-separation strategy in asynchronous mESCs allowed us to measure the levels of light ‘old’ and heavy, newly deposited methyl marks, and oxidation of those marks to hydroxymethylation, on both the parental and the newly replicated strands post-replication (Fig. 5a). By 8 h post-replication, EdU^+^ dsDNA contained light and heavy methylation in an approximate 50:50 ratio, indicating our timecourse captured the vast majority of restoration (Extended Data Fig. 5a). Intriguingly, at 12 h, one full cell cycle post-replication, EdU^+^ dsDNA contained more heavy methylation than light methylation, suggestive of de novo methylation (Extended Data Fig. 5b). As expected, most of the new, heavy methylation was deposited onto the newly synthesized (EdU^+^) strand (Fig. 5b and Extended Data Fig. 5c). This was also observed in a second cell line, NIH3T3 cells (Extended Data Fig. 5d). However, parental ssDNA unexpectedly contained small but increasing levels of heavy methylation throughout the timecourse, indicating deposition on both strands contributes to methylation dynamics in mESCs (Fig. 5c and Extended Data Fig. 5c). Importantly, overall methylation levels on parental ssDNA increased throughout the timecourse, indicating the heavy methylation accumulation was not solely attributable to turnover of methyl marks (Fig. 2d). Accumulation of heavy methylation was also observed in NIH3T3 cells (Extended Data Fig. 5e). Therefore, both the nascent and parental strands gain methylation in the wake of replication, and this is a general feature of methylation dynamics in both pluripotent and differentiated cells.

In our metabolic labelling approach, a 50:50 ratio of light:heavy modifications on gDNA can be used as a proxy to indicate complete symmetry of methylation or hydroxymethylation between complementary DNA strands. However, hydroxymethylation levels instead showed only negligible heavy levels in gDNA after one cell cycle in mESCs (Extended Data Fig. 5f). We observed the same difference in somatic NIH3T3 cells (Extended Data Fig. 5g). This, therefore, is a feature of hydroxymethylation generally, and is not unique to pluripotent cells, which are known to have substantially higher hydroxymethylation levels.

Post-replicative hydroxymethylation establishment is thought to occur primarily on the parental DNA strand^13^. iDEMS can test this by specifically measuring hydroxymethylation on the two DNA strands post-replication. We observed very low but increasing hydroxymethylation on the EdU^+^ ssDNA strand throughout our timecourse (Fig. 5d). Looking at parental ssDNA, we also observed accumulating hydroxymethylation over time, the majority of which was light (Fig. 5e). One full cell cycle post-replication, the parental strand still contained markedly more hydroxymethylation than the newly replicated strand (Extended Data Fig. 5h). Therefore, hydroxymethylation is never equally present on complementary DNA strands at any point in the cell cycle, and its distribution is continually biased towards the older, template DNA strand.

### Epigenetic crosstalk in nascent chromatin

**Extended Data Fig. 6:**
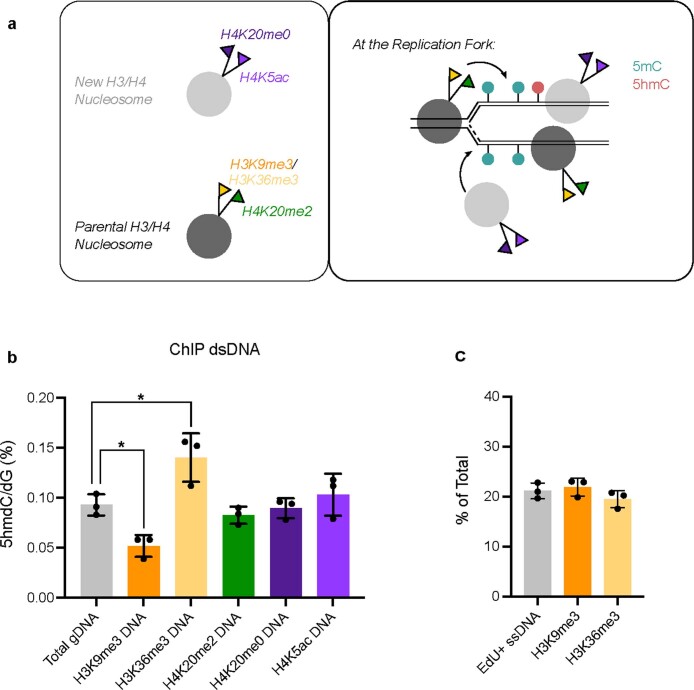
Extended Data Related to Fig. 6. **a**) Schematic showing histone PTM localization at the replication fork. **b**) Hydroxymethylation levels in H3K9me3-, H3K36me3-, H4K20me2-, H4K20me0-, and H4K5ac-associated DNA, compared to gDNA. **c**) Methylation levels in Fig. 5c (ChIP-iDEMS EdU+ ssDNA) shown as a percentage of methylation measured in the corresponding ChIP-MS dsDNA. EdU+ ssDNA shown as a percentage of gDNA. In b, c), data are presented as mean values +/− SD from three biological replicates. P-values calculated using one-way ANOVA, adjusting for multiple comparisons: *, p < 0.05; **, p < 0.01; ***; p < 0.001 ****, p < 0.0001. Exact adjusted p-values for b): total gDNA v H3K9me3 DNA: p = 0.0273; total gDNA v H3K36me3 DNA: p = 0.0112; total gDNA v H4K20me2 DNA: p = 0.8763; total gDNA v H4K20me0 DNA: p = 0.9986; total gDNA v H4K5ac DNA: p = 0.8763. Numerical source data are provided. Source data

Crosstalk between DNA methylation and various histone post-translational modifications (PTMs) has been well documented^34^. However, the nucleosomes on which histone PTMs reside present a barrier between DNA methyltransferases and DNA^7^. In DNA replication, nucleosome assembly is tightly coordinated with fork progression, and parental histone recycling and deposition of newly synthesized histones cumulatively restore nucleosome density on the new sister strands (Extended Data Fig. 6a). Parental and new histone H3–H4 tetramers bear distinct PTMs: parental histones carry a vast array of PTMs, including lysine trimethyl marks, and are near-universally marked with methylation on H4K20—mainly H4K20me2 (ref. ^29^). New tetramers are distinguished by H4K20me0 and acetylation at H4K5 and H4K12 immediately after deposition^29^.

**Fig. 6:**
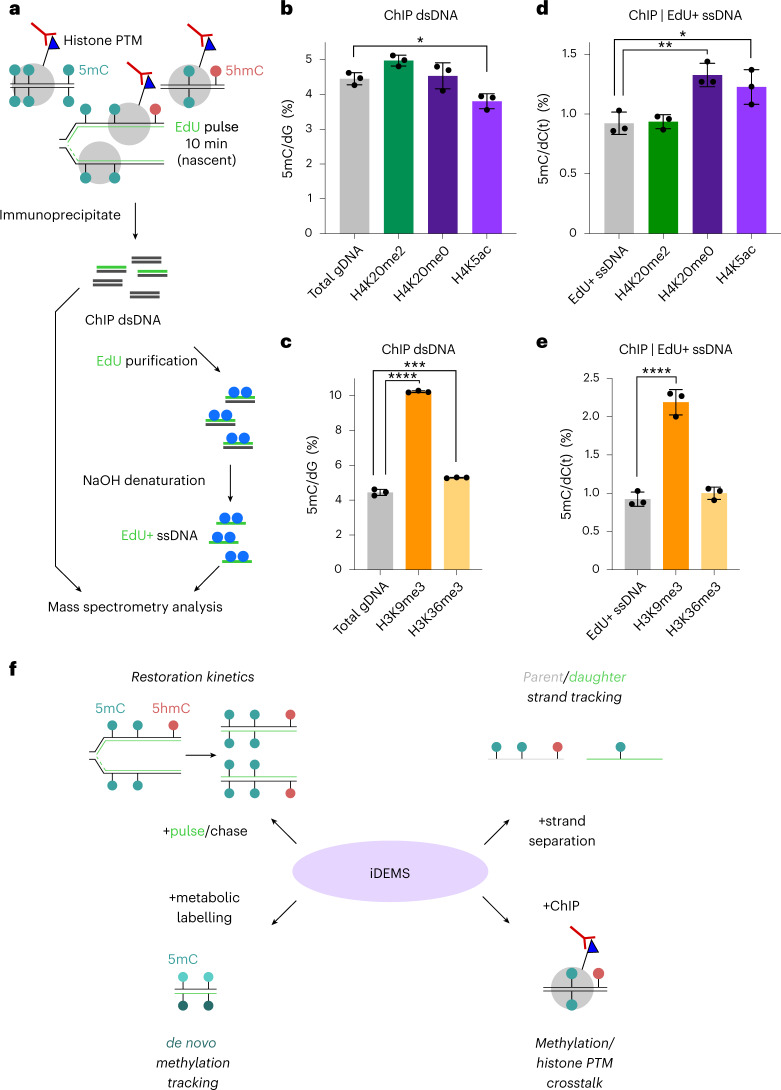
ChIP-iDEMS measures DNA modifications on histone PTM-associated DNA. **a**, Schematic of strategy for mass spectrometry analysis of EdU-labelled, immunoprecipitated DNA. **b**, Methylation levels in H4K20me2-, H4K20me0- and H4K5ac-associated DNA, compared with gDNA. **c**, Methylation levels in H3K9me3- and H3K36me3-associated DNA, compared with gDNA. **d**, Methylation levels in H4K20me2-, H4K20me0- and H4K5ac-associated EdU^+^ ssDNA, compared with EdU^+^ ssDNA. **e**, Methylation levels in H3K9me3- and H3K36me3-associated EdU^+^ ssDNA, compared with EdU^+^ ssDNA. **f**, iDEMS opens new avenues for understanding DNA modifications. In **b**–**e**, data are presented as mean ± s.d. from three biological replicates. *P* values calculated using one-way ANOVA, adjusting for multiple comparisons: *P < 0.05; **P < 0.01; ***P < 0.001; ****P < 0.0001. Exact adjusted *P* values for **b**: total gDNA versus H4K20me2 DNA: *P* = 0.0758; total gDNA versus H4K20me0 DNA: *P* = 0.9522; total gDNA versus H4K5ac DNA: *P* = 0.0298. Exact adjusted *P* values for **c**: total gDNA versus H3K9me3 DNA: *P* < 0.0001; total gDNA versus H3K36me3 DNA: *P* = 0.0001. Exact adjusted *P* values for **d**: EdU^+^ ssDNA versus H4K20me2 DNA: *P* = 0.9987; EdU^+^ ssDNA versus H4K20me0 DNA: *P* = 0.0035; EdU^+^ ssDNA versus H4K5ac DNA: *P* = 0.0175. Exact adjusted *P* values for **e**: EdU^+^ ssDNA versus H3K9me3 DNA: *P* < 0.0001; EdU^+^ ssDNA versus H3K36me3 DNA: *P* = 0.6637. Numerical source data are provided. Source data

To investigate whether DNA methylation restoration differs on the basis of chromatin context, we immunoprecipitated mononucleosomes carrying histone PTMs of interest from EdU-pulsed cells and analysed both the bulk immunoprecipitated DNA and the EdU^+^ ssDNA fraction by mass spectrometry (ChIP-iDEMS) (Fig. 6a). While H4K20me0- and H4K20me2-associated DNA had largely similar methylation levels to gDNA, H4K5ac-associated DNA bore significantly less methylation (Fig. 6b), probably because genome wide this mark is associated with active regulatory elements^35^. Looking specifically at methylation levels in DNA immunoprecipitated with known crosstalk marks, DNA associated with H3K9me3, a marker of constitutive heterochromatin^3^, was especially enriched in methylation, with one in ten cytosines methylated (Fig. 6c). In contrast, H3K36me3-associated DNA, which overlaps with DNA methylation in gene bodies^1^, showed only slightly higher enrichment than gDNA (Fig. 6c). Conversely, hydroxymethylation was relatively depleted on H3K9me3 DNA and enriched on H3K36me3 DNA (Extended Data Fig. 6b). By quantifying modifications on immunoprecipitated DNA by mass spectrometry, ChIP-iDEMS helps contextualize analyses of DNA–histone crosstalk conventionally done with sequencing approaches.

We next turned to the EdU^+^ ssDNA fraction of our datasets. We focused on nascent chromatin, where new and parental histones can be distinguished by H4K20 methylation status. Surprisingly nucleosomes assembled from de novo deposited histones, marked by H4K20me0 and H4K5ac, carried more DNA methylation than nucleosomes assembled from recycled old histones (Fig. 6d). Therefore, although in nascent chromatin only parental histones carry the trimethyl marks associated with DNA–histone crosstalk, DNA wrapped around new histones has significantly higher methylation.

The trends observed in bulk immunoprecipitates of H3K9me3 and H3K36me3 were mirrored on nascent chromatin (Fig. 6e). Though H3K9me3-associated DNA had more than twice as much methylation as EdU^+^ ssDNA controls, this corresponded to approximately 20% of the level measured in the bulk H3K9me3 pulldown, mirroring the rate of methylation genome wide seen in EdU^+^ ssDNA (Extended Data Fig. 6c). On the basis of this, we conclude that, though the rate of re-methylation immediately after replication is higher in H3K9me3-marked regions compared with the average rate genome wide, in nascent chromatin its methylation level is proportionate to that seen in steady state.

## Discussion

iDEMS is a mass-spectrometry-based technology that provides an important orthogonal method to bisulfite sequencing-based approaches, providing global information and avoiding any biases that may arise in methodologies that require amplification. We demonstrate that iDEMS can be combined with additional techniques, such as metabolic labelling and ChIP, to reveal important insights into DNA methylation metabolism and DNA methylation–histone modification crosstalk (Fig. 6f). iDEMS is therefore a dynamic and informative tool for addressing important questions in epigenome maintenance and DNA modification biology.

By directly quantifying DNA modifications on replicated DNA, iDEMS definitively resolves DNA methylation and hydroxymethylation kinetics after DNA replication. Our data revealed that, in the immediate wake of the replication fork, the nascent chromatin landscape is primarily hemi-methylated, with the newly replicated strand bearing only 20–30% of genomic methylation levels. Methylation levels steadily increased up to 4 h after replication, the time at which methylation levels on replicated DNA and the bulk gDNA were equal. This indicates that this process proceeds at a steady, and slow, pace. This result is consistent with the kinetics defined via repli-bisulfite sequencing (Repli-BS-seq)^31^, which were also generated in asynchronous pluripotent cells but used bromodeoxyuridine rather than EdU to label replicated DNA. Repli-BS-seq uses immunoprecipitation of labelled DNA fragments rather than Click-IT to isolate labelled DNA, necessitating a longer labelling time (1 h). This means repli-BS-seq lacks the resolution to track early methylation restoration events, and cannot differentiate replication fork-coupled from replication fork-uncoupled maintenance.

Both iDEMS and Hammer-seq^15^ utilize EdU to track restoration kinetics. iDEMS-defined kinetics differ, however, from the restoration kinetics seen by Hammer-seq^15^, which compared hairpin-sequenced parent and daughter strands in synchronized HeLa cells and asynchronous mESCs and in both found over 50% of methylation restored within 4 min of replication. By 30 min post-replication, over 80% of methylation in HeLa cells was restored by Hammer-seq; in our data we see approximately 40% of methylation restored by this time. Hammer-seq provides important information on relative maintenance rates across the genome^15^, but it remains unclear why it appears to overestimate methylation restoration kinetics. This could be linked to amplification and sequencing biases that can overestimate methylation levels, or a consequence of the fact that we measure methylation in embryonic and primary cells, while Hammer-seq was performed in a cancer cell line^15^. Given that the kinetics measured by Hammer-seq also differ from those of other sequencing approaches^30,31^, complementary and direct assays such as iDEMS are important in defining absolute methylation maintenance rates globally.

Given that we achieve over 99% purity of pulled-down DNA in our iDEMS protocol, do not subject samples to any conversion that affects cytosine residues, analyse only cytosine and guanine nucleosides (eliminating both thymidines and any thymidine analogues from analysis), and that our protocol contains no amplification step that could skew signal, we are confident that iDEMS reliably tracks methylation maintenance kinetics. With low-input nucleoside analysis by mass spectrometry becoming more and more common, iDEMS is readily employable by anyone with access to high-sensitivity mass spectrometers. Further optimization of EdU-based sequencing approaches may improve the current discrepancies without sacrificing the high resolution facilitated by short labelling times. Alternatively, sequencing-based approaches that do not require amplification such as Nanopore sequencing^36,37^ could be developed to address locus-specific differences in methylation restoration.

The methylation kinetics we observed in our global timecourse were replicated in our early- and late-S timecourses. A consequence of such a slow rate of re-methylation would be incomplete restoration of the methylome at the time of mitosis. This prediction was supported by our flow cytometry data, which showed cells in our late-S timecourse were primarily in G1 phase 4 h post-labelling. Consistently, we found small but real differences in the methylome pre- and post-mitosis, and revealed that DMRs were disproportionately hypermethylated in G1 in particular in late-replicating regions. These results corroborate conclusions from Hammer-seq^15^ and observations that regions of DNA hypomethylation in cancer show high overlap with late-replication domains^4,5,28^. Similar observations have been made in studies of DNA methylation drift in ageing^6,38,39^. Both cancerous and ageing cells have undergone numerous mitotic divisions; a slow pace of methylation maintenance would provide the molecular mechanism by which DNA methylation is lost over the course of many cell divisions. Intriguingly, we also identified a number of DMRs with higher methylation in the G2/M population. These may represent aberrant methylation that is removed, possibly via oxidation and recruitment of base excision-repair machinery^10^, as part of chromatin maturation.

By combining iDEMS with metabolic labelling and a strand-separation strategy, we documented the dynamics of new and old methylation marks post-replication. Unexpectedly, methylation levels on both the parental and newly replicated strands increase post-replication, as a consequence of newly deposited methyl marks. This suggests that DNA methylation maintenance is a less precise process than the copy–paste model has traditionally depicted, with inexact targeting of methyltransferases acting to maintain overall methylation levels rather than preserving the fidelity of the methylation landscape between cell divisions.

DNA hydroxymethylation, which has never been studied directly on post-replicative DNA before, showed perpetual asymmetry between the parental and newly replicated strands. This was due to the persistent gain of hydroxymethylation on the parental strand. This makes hydroxymethylation a mark that continuously distinguishes the template strand. Our finding is consistent with earlier work that measured hydroxymethylation on global DNA^13^, which proposed that oxidation was sequestered to the parental strand post-replication. It has been proposed that higher hydroxymethylation marks the ‘immortal strand’ during asymmetric stem cell divisions^40^, and our findings are consistent with single-cell, strand-specific hydroxymethylation sequencing that has shown hydroxymethylation can be used as a proxy for DNA strand age^14^. This distinctive localization also makes hydroxymethylation a potential player in processes such as mismatch repair, though due to the rarity of this modification such a function would be restricted to specific genomic regions such as enhancers and promoters. Several proteins that specifically recognize hydroxymethylation over methylation are involved in mismatch repair^41^, and there is evidence that hydroxymethylation can recruit specialized repair complexes to complete demethylation^42^. In future work, it will be interesting to profile co-localization of hydroxymethylation with players in post-replication DNA repair pathways.

ChIP-iDEMS is a mass-spectrometry-based approach to studying crosstalk between DNA methylation and histone modifications. This method revealed unexpected insights into both the roles of histone modifications in nascent chromatin methylation and DNA methylation–histone PTM crosstalk more generally. ChIP-MS of the parental histone mark H4K20me2 and the naïve histone marks H4K20me0 and H4K5ac showed higher methylation levels associated with H4K20me2, while ChIP-iDEMS showed the opposite trend. It has been proposed that, during the replication fork-coupled phase of methylation maintenance, the methyltransferase machinery exploits the brief interval between DNA synthesis and histone deposition to access and methylate DNA^15^. Our ChIP-iDEMS data support this hypothesis: since naïve histone deposition is thought to be slightly slower than parental histone recycling^29^, the EdU-labelled DNA immunoprecipitated with H4K20me0 or H4K5ac would be comparatively accessible for a longer period of time, resulting in higher rates of methylation immediately following replication. It could also be that these increased levels reflect the slight bias of naïve histone deposition toward the lagging strand seen in mESCs^43^, since the lagging strand has previously been shown to re-methylate at a faster rate than the leading strand^15^. This phenomenon could also be explained in part by UHRF1 targeting to LIG1 (ref. ^17^), which is specific to the lagging strand. Implementing ChIP-iDEMS technology on replisome and methylation mutants will help discriminate between these possibilities.

iDEMS will be useful in profiling methylation and hydroxymethylation dynamics in different cellular contexts, including development, ageing and tumour evolution. By providing an orthogonal approach to the often-used bisulfite sequencing, iDEMS benefits the DNA methylation field and has the potential to challenge conclusions drawn from a single method. Compared with sequencing data, mass spectrometry provides a simple, fast readout, and iDEMS could therefore be useful where efficiency is key, such as in medical settings and drug discovery studies. We envision that in addition to the methodologies described here, iDEMS could be combined or augmented with additional technologies, such as sequence-specific capture to analyse DNA modifications in specific loci or repli-seq^44^ to further parse restoration kinetics within replication domains. Our findings using iDEMS complement existing genetic analyses of post-replicative methylation kinetics^15^ and add an important tool to the suite of technologies investigating epigenome stability.

## Methods

A step-by-step iDEMS protocol, including options for stable isotope labelling with amino acids in cell culture (SILAC)-iDEMS and chromatin immunoprecipitation (ChIP)-iDEMS, can be found at Protocol Exchange^46^.

### Cell Culture

MESC lines used in this study are E14 background and were gifted by Kristian Helin (commercial source: Mutant Mouse Regional Resource Center at UC Davis) and Joshua Brickman (derived by Jan Ure while at the University of Edinburgh). E14 mESCs were grown on plates coated with 0.2% gelatin (Sigma, G9391). Cells were thawed and cultured in light mESC media: Dulbecco’s modified Eagle medium (DMEM; Gibco, 31966-021) supplemented with 15% dialysed foetal bovine serum (Gibco, 26400-036), 1× penicillin–streptomycin (Gibco, 151400122), 1× GlutaMAX (Gibco, 35050061), 1× non-essential amino acids (Gibco, 11140050), 1× sodium pyruvate (Gibco, 11360-070), 11140050), 0.4 μM β-mercaptoethanol (Sigma, M3148) and custom-made leukaemia inhibitory factor. Cells were incubated at 37 °C with 5% CO_2_.

NIH3T3 cells used in this study were gifted by Berthe Marie Willumsen (derived originally by the Doug Lowy lab). NIH3T3 cells were thawed and cultured in light NIH3T3 medium: DMEM medium (Gibco, 31966-021) supplemented with 10% dialysed FBS (Gibco, 26400-036) and 1× penicillin–streptomycin (Gibco, 151400122). For SILAC experiments, the same recipes as above were used, with the following modifications: DMEM without methionine or cysteine (Gibco, 21013-024) was used in place of DMEM, and supplemented with 30 mg l^−1^ l-methionine (methyl-^13^C,d_3_) (Sigma, 299154) and 63 mg l^−1^ cysteine hydrochloride (Sigma, 30120).

*Drosophila* S2 cells used in this study were obtained from the *Drosophila* Genomics Resource Center. Cells were grown in suspension in spinners in M3 + BPYE medium: Shields and Sang M3 Insect Medium (Sigma, S-8398), KHCO_3_ (Sigma, 12602), yeast extract (Sigma, Y-1000), bactopeptone (BD, 211705), 10% heat-inactivated foetal calf serum (FCS; GE Hyclone, SV30160.03) and 1× penicillin–streptomycin (Gibco, 151400122). Cells were incubated at 25 °C with 5% CO_2_.

For asynchronous iDEMS in mESCs, 1 × 10^7^ cells were seeded in 15 cm dishes 1 day before EdU labelling and processing, four dishes per timepoint. For synchronized iDEMS in mESCs, 1 × 10^7^ cells were seeded in 15 cm dishes 1 day before EdU labelling and processing, two dishes per timepoint. For asynchronous iDEMS in NIH3T3s, 5 × 10^6^ cells were seeded in 15 cm dishes 1 day before EdU labelling and processing, four dishes per timepoint. In asynchronous SILAC experiments, cells were seeded in light medium and switched to heavy medium 4 h before EdU labelling. For ChIP-iDEMS, 4 × 10^6^ cells were seeded in 15 cm dishes 2 days before EdU labelling and processing, six dishes per timepoint. For EM-seq, mESCs were grown to 70–80% confluence before EdU labelling and processing, one dish per timepoint. Where relevant, control plates were seeded in parallel using the same seeding density as the iDEMS plates and collected in parallel for flow cytometry analysis.

### Cell synchronization

Cells were grown in light medium and arrested at the G1/S boundary by addition of 2 mM thymidine (Sigma, T9250) for 12 h. Cells were then washed twice in 1× phosphate-buffered saline (PBS) and released into heavy medium containing 24 μM deoxycytidine (Sigma, D0776). To label early-replicating DNA, cells were pulsed with 20 μM 5-ethynyl-2′-deoxyuridine (EdU; Invitrogen, A10044) 1.5 h after release; to label late-replicating DNA, cells were pulsed with 20 μM EdU 4.5 h after release.

### DNA labelling

mESC samples were labelled with medium containing EdU at a final concentration of 20 μM for 10 min. NIH3T3 samples were labelled with EdU at a final concentration of 20 μM for 1 h. The differing labelling times reflect the difference in the proportion of cells in S phase between these cell types. Following the labelling, nascent samples were immediately taken and processed. All mature samples were washed twice in 1× PBS and further incubated in fresh medium containing 10 μM thymidine (Sigma, T9250) for the appropriate time interval before collection.

### iDEMS

After EdU labelling and, if relevant, chasing the EdU pulse for the desired interval, media was immediately discarded and cells were washed twice in ice-cold 1× PBS. Ice-cold 70% ethanol was then added and plates were kept at −20 °C for at least 1 h. Cells were scraped in the ethanol, pipetted into Falcon tubes (combining dishes from the same timepoint), and spun at 400*g* for 5 min. gDNA was isolated from samples using the Zymo Quick-DNA Midiprep kit (Zymo, D4075). Up to 60 μg DNA per sample was sonicated in 10 μg aliquots to 300 bp using a Covaris S220 or Covaris E220. If needed, approximately 400 ng of sonicated gDNA was put aside as a gDNA control.

To perform Click-IT, up to 10 μg sonicated DNA was incubated for 30 min at room temperature (20–25 °C) with the following conditions (final volume: 200 μl): 1× Click-IT buffer (Click-iT EdU Alexa Fluor 488 Imaging Kit, Thermo Fisher, C10337), 1 mM picolyl-azide-PEG4-biotin (Jena Bioscience, CLK-1167-100), 0.1 mM CuSO4 (from Click-iT kit), 0.5 mM THPTA (Sigma, 762342) and 10 mM sodium ascorbate (from Click-iT kit). DNA was then purified using double-size selection with Agencourt AMPure XP beads (Beckman Coulter, A63881); first a 0.5:1 bead:sample ratio, then a 3:1 ratio. DNA was eluted in 250 μl EB buffer.

To capture biotinylated fragments, 10 μl Dynabeads MyOne Streptavidin T1 beads (Invitrogen, 65602) per sample were washed three times with 1× B&W buffer (5 mM Tris–HCl pH 7.5, 0.5 mM ethylenediaminetetraacetic acid (EDTA), 1 M NaCl and 0.05% Tween-20) and resuspended in 2× B&W buffer at a volume equal to the volume of biotinylated DNA (final volume: 500 μl). Streptavidin beads and DNA samples were then mixed and incubated for 30 min at room temperature with side rotation. Following incubation, beads were then pelleted on a magnetic rack. Beads bound to biotinylated DNA were washed six times with 1× B&W buffer, twice with 1× TE with 0.05% Tween-20 and once with 10 mM Tris–HCl pH 7.5. Beads were then resuspended in 10 μl EB buffer.

For analysis of EdU^+^ dsDNA only, the Click-IT, DNA purification, and streptavidin pulldown steps were done on 3 × 10 μg sonicated DNA aliquots per sample and the DNA-bound beads were pooled for LC/MS. For analysis of EdU^+^ dsDNA, EdU^+^ ssDNA and parental ssDNA from the same sample, the Click-IT, DNA purification and streptavidin pulldown steps were done on 6 × 10 μg sonicated DNA aliquots per sample and the DNA-bound beads were pooled. One-third of each sample was put aside as the EdU^+^ dsDNA sample. The remaining sample was then resuspended in 100 μL 100 mM NaOH with 0.05% Tween-20, incubated at room temperature for 1 min, and pelleted on a magnetic rack. The supernatant was transferred to a new tube as the parental ssDNA sample. This alkaline wash was repeated twice, for a total of three washes, each time transferring the supernatant to the parental ssDNA tube. The streptavidin beads, now bound to the EdU^+^ ssDNA sample, were washed twice with 1× B&W buffer, twice with 1× TE with 0.05% Tween-20 and once with 10 mM Tris–HCl pH 7.5. Beads were then resuspended in 10 μl EB buffer. The parental ssDNA sample was purified with Agencourt AMPure XP beads (Beckman Coulter, A63881) using a 1.8:1 bead ratio and eluted in 10 μl high-performance liquid chromatography mass spectrometry grade water (Fisher, 10777404). Samples were then processed for LC/MS analysis.

### ChIP-iDEMS

After EdU labelling, medium was immediately discarded and cells were washed in ice-cold 1× PBS. Cells were scraped, pipetted into Falcon tubes (combining dishes from the same timepoint) and spun at 300*g* for 10 min at 4 °C. Cell pellets were resuspended in Buffer A (10 mM HEPES pH 7.9, 10 mM KCl, 1.5 mM MgCl_2_, 0.34 M sucrose and 10% glycerol) supplemented with inhibitors (1 mM phenylmethylsulfonyl fluoride (PMSF), 1 μg ml^−1^ leupeptin, 1 μg ml^−1^ pepstatin, 1 μg ml^−1^ aprotinin and 1 μg ml^−1^ trichostatin A), transferred to low-binding 1.5 ml tubes, and pelleted at 300*g* for 5 min at 4 °C. Pellets were resuspended in Buffer A supplemented with inhibitors and lysed by addition of 10% Triton-X 100 to a final concentration of 0.1% and gently inverted. Tubes were incubated horizontally on ice in a cold room for 7 min, and nuclei were pelleted at 300*g* for 5 min at 4 °C. Nuclei were washed twice more in Buffer A supplemented with inhibitors, pelleting at 300*g* for 5 min at 4 °C between washes. Following final supernatant aspiration, nuclei were resuspended in Buffer A supplemented with inhibitors. Suspension (2 μl) was taken for nuclei counting, and the remaining suspension was flash-frozen in 500 μl aliquots with liquid nitrogen and stored at −80 °C until MNase digestion.

To digest chromatin, nuclei were thawed for 5 min at 30 °C with 300 rpm on a thermomixer. CaCl_2_ (5 μl 100 mM) was added to each 500 μl nuclei suspension and mixed by inversion. MNase (1 μl; Warthington, 50 U μl^−1^) was added per 2.5 × 10^7^ nuclei, mixed by inversion, and incubated at 30 °C for 20 min at 300 rpm on a thermomixer. To stop digestion, tubes were immediately placed on ice. Then 10 μl of a pre-mixed 1:1 solution of 0.1 M egtazic acid pH 8.0 and 0.5 M EDTA pH 8.0 was added to each tube and mixed by inversion. Triton-X 100 (5 μl, 10%) and 75 μl 2 M KCl were added to each tube and mixed by inversion. PMSF (5 μl, 1 mM), 0.5 μl 1 μg ml^−1^ leupeptin, 0.5 μl 1 μg ml^−1^ pepstatin, 0.5 μl 1 μg ml^−1^ aprotinin and 0.5 μl 1 μg ml^−1^ trichostatin A were added to each tube and mixed by inversion. Digested chromatin was elutriated ten times through a 21-gauge needle attached to a 1 ml syringe in a cold room and rotated at 20 rpm at 4 °C to release chromatin. To separate the soluble chromatin fraction, samples were spun at 14,000*g* for 10 min at 4 °C and the supernatants transferred to new tubes. Native chromatin was immediately used in immunoprecipitation.

To quantify chromatin and prepare control gDNA and EdU^+^ ssDNA samples for LC/MS, 10 μl of supernatant was transferred to a new 1.5 ml tube and mixed with 90 μl of TE pH 8.0 and 2.5 μl of 20% sodium dodecyl sulfate (SDS). Samples were incubated for 15 min at 37 °C with 300 rpm on a thermomixer, purified using the Qiagen QIAquick PCR Purification Kit (Qiagen, 28104), eluted in LC/MS-grade water (Fisher, 10777404) and quantified. Ten per cent of DNA was set aside as gDNA controls for LC/MS; the remaining 90% was subjected to iDEMS to purify EdU^+^ ssDNA controls. To confirm digestion to mononucleosomes, an aliquot of purified DNA was additionally run on a BioAnalyzer (Agilent).

For each ChIP, 50 μg of chromatin (measured as DNA concentration) and the indicated amount of antibody (5 μg, H4K20me2 (Diagenode, C15200205); 10 μg, H4K20me0 (Abcam, 227804); 10 μg, H4K5ac (Abcam, ab51997); 5 μg, H3K36me3 (Abcam ab9050); 6.4 μg, H3K9me3 (Abcam, ab176916)) was used. The volume was adjusted to 500 μl with Buffer D (20 mM HEPES pH 7.9, 0.2 mM EDTA pH 8.0, 250 mM KCl, 20% glycerol and 0.2 Triton-X 100). All antibodies used are commercially available and have been validated by the manufacturers for ChIP. H4K20me0, H4K5ac and H3K9me3 antibodies have reactivity in mouse validated by the respective manufacturers; H4K20me2 and H3K36me3 antibodies have reactivity in mouse as previously reported^43^. A no antibody control was included with each ChIP. Samples were incubated overnight on a rotating platform in a cold room. Immunoprecipitated fragments were captured using 150 μl anti-rabbit (Invitrogen, 11203D) or anti-mouse (11202D) immunoglobulin G Dynabeads, washed three times in Buffer D before use. Samples were incubated with immunoglobulin G Dynabeads for 2–3 h on a rotating platform at 20 rpm at 4 °C.

Immunoprecipitated chromatin fragments were pelleted on a magnetic rack and washed three times with 500 μl of low salt washing buffer (20 mM Tris–HCl pH 8.0, 2 mM EDTA, 150 mM NaCl, 1% Triton-X 100 and 0.1% SDS) and three times with 500 μl of high salt washing buffer (20 mM Tris–HCl pH 8.0, 2 mM EDTA, 500 mM NaCl, 1% Triton-X 100 and 0.1% SDS), changing to new prechilled 1.5 ml low-binding tubes after the first wash only. Fragments were eluted in 200 μl ChIP elution buffer (20 mM Tris–HCl pH 8.0, 10 mM EDTA and 1% SDS) for 30 min at 37 °C, purified with the Qiagen QIAquick PCR Purification Kit (Qiagen, 28104) and eluted into 50 μl of EB (10 mM Tris–HCl pH 8.5). All buffers for chromatin preparation and ChIP were supplied with 1 mM PMSF, 1 μg ml^−1^ leupeptin, 1 μg ml^−1^ pepstatin, 1 μg ml^−1^ aprotinin, and 1 μg ml^−1^ trichostatin A.

DNA was then purified using double-size selection with Agencourt AMPure XP beads (Beckman Coulter, A63881); first a 0.8:1 bead:sample ratio, then a 3:1 ratio. DNA was eluted in 85 μl LC/MS-grade water (Fisher, 10777404). ChIP DNA (5 μl per sample) was put aside as ChIP dsDNA samples for LC/MS. The remaining 80 μl was subjected to the iDEMS protocol, except that a 2:1 single-size selection was done during post-Click-IT DNA purification with AMPure beads, and utilizing strand separation to purify EdU^+^ ssDNA samples for LC/MS.

### 5mdC and 5hmdC quantification by LC–MS/MS

A minimum of 1 ng of DNA was digested to nucleosides overnight at 37 °C using a nucleoside digestion mix (NEB, M0649) previously purified using 10 kDa Centrifugal Filter Units (Millipore, MRCPRT010). The volume of digestion mix injected for quantification was experimentally tested to use a similar amount of nucleosides between samples and within the range of quantification. Digestion mix without DNA at the same dilution was always used as a control for potential external contamination. The nucleosides were separated on an Agilent RRHD Eclipse Plus C18 2.1 × 100 mm 1.8 μm column using the HPLC 1290 system (Agilent) and mobile phase A: 0.1% formic acid in 100% water and B: 0.1% formic acid in 80% methanol (chromatographic details in Supplementary Table 2). Quantification was carried out in an Agilent 6490 triple quadrupole mass spectrometer in multiple reaction monitoring mode, by monitoring specific transition pairs (Supplementary Tables 3 and 4). Mass spectrometry data were measured and analysed using Agilent MassHunter acquisition software for LC/MS systems (v10.0). To calculate the concentrations of individual nucleosides, standard curves were generated from standards (dC, dG and 5hmdC from Berry and Associates; 5mdC from CarboSynth) (Supplementary Fig. 1). Mass spectrometry assessment of nucleoside standards and the adducts generated are shown in Supplementary Fig. 2. Samples and standard curve points were spiked with isotope-labelled synthetic nucleosides (^13^C^15^N-dC and ^13^C^15^N-dG were purchased from Silantes, and d_2_^15^N_2_-5hmdC was obtained from T. Carell, Center for Integrated Protein Science at the Department of Chemistry, Ludwig-Maximilians-Universität München, Germany). Final measurements were normalized by dividing by the dG level measured for the same sample for dsDNA samples or by total dC levels (dC + 5mdC + 5hmdC) for ssDNA samples. Peaks were quantified if falling within the linear range, with a signal-to-noise ratio above 10 and accuracy of at least 20% of calculated versus expected concentration of the standard curve. Limit of quantification was 0.025 fmol for 5mdC, 0.005 fmol for 5hmdC and 0.5 fmol for dC and dG. Limit of detection (LOD) was 0.005 fmol for 5mdC and 5hmdC, and 0.1 fmol for dC and dG. Limit of quantification or LOD for a specific datapoint was calculated as a percentage of dG or dC(t) for that sample.

### Flow cytometry

To sort G1 and G2M cell populations, mESCs were grown to 70–80% confluence on a coated 15 cm dish and labelled with EdU at a final concentration of 20 μM for 10 min. Immediately after labelling, cells were collected with trypsin. After fixation with ice-cold 70% ethanol, cells were incubated at 4 °C for a minimum of 1 h. Cells were then spun down at 500*g* for 5 min at room temperature and permeabilized in 1× PBS with 1% FCS and 0.25% Triton-X 100 for 10 min at room temperature. Cells were spun down at 500*g* for 5 min at room temperature and resuspended in 1× PBS with 1% FCS for counting. Five aliquots of 5 × 10^6^ cells and three aliquots of 1 × 10^6^ cells (for single colour controls) were transferred to new tubes and spun down at 500*g* for 5 min at room temperature. The appropriate tubes were resuspended in Click-IT reaction mix with the following conditions: 1× Click-IT buffer (Click-iT EdU Alexa Fluor 488 Imaging Kit, Thermo Fisher, C10337), 2 mM CuSO_4_ (from Click-iT kit), 10 mM sodium ascorbate (from Click-iT kit), and Alexa Fluor azide (1:1,000 dilution, prepared as directed in Click-iT EdU Alexa Fluor Imaging Kit). Cells were incubated for 30 min at room temperature in the dark, then spun down and the appropriate tubes were resuspended in 1× PBS with propidium iodide (10 μg ml^−1^ final concentration) and RNAse A (20 μg ml^−1^ final concentration). Cells were incubated overnight at 4 °C in the dark before washing and sorting on a BD Aria III flow cytometer. Flow cytometry profiles were analysed by FlowJo 10.8 software.

For confirmation of cell synchronization and release, mESCs were synchronized and released as described above. Following fixation with ice-cold 70% ethanol, cells were permeabilized and labelled with propidium iodide as described above before analysing on a LSR Fortessa X20 flow cytometer.

### EM-seq

Libraries were generated in biological triplicate in G1 and G2M sorted cell populations using the EM-seq method^33^ (NEB, E7120S). DNA (125 ng per sample) was mixed with 1 pg pUC19 DNA and 20 pg lambda DNA and sonicated to 200 bp using a Covaris E220. *Drosophila* DNA was sonicated in parallel, 7.5 pg was added to sonicates as an additional conversion control, and polymerase chain reaction (PCR)-grade water was added to a final volume of 50 μl. NEBNext Ultra II End Prep Reaction Buffer (7 μl) and 3 μl NEBNext Ultra II End Prep Enzyme Mix was added to each sample, mixed and incubated at 20 °C for 30 min followed by 65 °C for 30 min in a thermocycler with a lid heated to 65 °C. On ice, 2.5 μl NEBNext EM-seq Adaptor, 1 μl NEBNext Ligation Enhancer and 30 μl NEBNext Ultra II Ligation Master Mix was added to each sample, mixed and incubated at 20 °C for 60 min in a thermocycler with an opened lid. Samples were purified using NEBNext Sample Purification Beads, 1.8:1 bead:sample ratio, and eluted in 28 μl Elution Buffer. Freshly prepared TET2 Reaction Buffer (10 μl), 1 μl Oxidation Supplement, 1 μl DTT, 1 μl Oxidation Enhancer and 4 μl TET2 enzyme were added to each sample and mixed. Freshly diluted 400 μM Fe(II) Solution (5 μl) was added to each sample, mixed and incubated at 37 °C for 60 min in a thermocycler with a lid heated to 45 °C. On ice, 1 μl Stop Reagent was added to each sample, mixed and incubated at 37 °C for 30 min in a thermocycler with a lid heated to 45 °C. Samples were purified using NEBNext Sample Purification Beads, 1.8:1 bead:sample ratio, and eluted in 16 μl Elution Buffer. Samples were denatured by adding 4 μl freshly prepared 0.1N NaOH, mixing and incubating at 50 °C for 10 min in a thermocycler with a lid heated to 60 °C. On ice, 68 μl PCR-grade water, 10 μl APOBEC Reaction Buffer, 1 μl bovine serum albumin and 1 μl APOBEC enzyme were added to each sample, mixed and incubated at 37 °C for 3 h in a thermocycler with a lid heated to 45 °C. Samples were purified using NEBNext Sample Purification Beads, 1:1 bead:sample ratio, and eluted in 20 μl Elution Buffer. Libraries were amplified by adding 5 μl EM-seq Index Primer and 25 μl NEBNext Q5U Master Mix to each sample, mixing and cycling with the following conditions: 98 °C, 30 s; five cycles of: 98 °C, 10 s; 62 °C, 30 s; 65 °C, 60 s; followed by a 65 °C, 5 min final extension. Libraries were purified in two rounds using NEBNext Sample Purification Beads, first round 0.9:1 bead:sample ratio; second round 1:1 bead:sample ratio, and eluted in 10 μl Elution Buffer. Libraries were checked for quality control by BioAnalyzer and Qubit quantification before sequencing.

### Library sequencing and processing

EM-seq libraries were sequenced 75 bp paired-end on an Illumina NextSeq 500. Fastq files were generated using bcl2fastq v2.19.1. FastQC v0.11.7 (http://www.bioinformatics.babraham.ac.uk/projects/fastqc) was used for quality control metrics, and multiqc v1.7 (ref. ^47^) for reporting. Reads were trimmed with trim_galore 0.6.4 (https://github.com/FelixKrueger/TrimGalore), mapped to the GRCm38/mm10 mouse reference genome and control genomes with bowtie2 using Bismark v0.22.1 (ref. ^48^) (bismark -N 1 -L 20 -X 1000), and deduplicated with deduplicate_bismark. Reads with MAPQ < 20 and PCR duplicates were discarded, as were reads found in outlier regions with a raw read count >10 above the median when the genome was partitioned into 25 kb windows. Remaining reads were used in downstream analyses.

### Sequencing analysis

All quantitations were done over 100 CpG windows, with a minimum of one in five CpGs being observed to include the window in analysis. When comparing datasets, a given window required a minimum of one in five CpGs being observed in all compared datasets to be included in analysis. DMRs were annotated using HOMER (v4.11)’s AnnotatePeaks function^49^. Gene Ontology analysis was done using GREAT (v4.0.4) (ref. ^50^) with default parameters. Replication domain definitions were taken from ref. ^45^. Analyses and figure generation were done in Seqmonk v1.47.1, RStudio v1.3.1093, and GraphPad Prism v9.2.0.

### Statistics and reproducibility

DMRs were identified using logistic regression, *P* < 0.05, adjusting for multiple comparisons. iDEMS statistics were computed using one-way analysis of variance (ANOVA), adjusting for multiple comparisons. Adjusted *P* values are annotated according to the following criteria: *, P < 0.05; **, *P* < 0.01; ***; *P* < 0.001 ****, *P* < 0.0001. No statistical method was used to pre-determine sample size. Sequencing experiments were designed to meet or exceed ENCODE standards^51^. For mass spectrometry experiments, our sample size (*n* = 3 for all experiments used in statistical comparisons) was sufficiently powered to detect significance at *P* < 0.05. No data were excluded from the analyses. During timecourse sample preparation, dishes were only labelled from their time of collection to ensure equal treatment of all samples before collection and random allocation of dishes into each timepoint. Following sample collection, samples were placed in groups on the basis of treatment: timepoint, DNA strand (stranded data), cell population (EM-seq data) or immunoprecipitated histone PTM (ChIP-iDEMS data). The investigators were not blinded to allocation during experiments and outcome assessment because no manual quantifications were performed.

All experiments have been performed in biological triplicate unless specified in the legends.

### Reporting summary

Further information on research design is available in the Nature Portfolio Reporting Summary linked to this article.

## Online content

Any methods, additional references, Nature Portfolio reporting summaries, source data, extended data, supplementary information, acknowledgements, peer review information; details of author contributions and competing interests; and statements of data and code availability are available at 10.1038/s41556-022-01048-x.

## Supplementary information

Supplementary Information Supplementary Figs. 1 and 2.

Reporting Summary

Supplementary Table 1 Supplementary Table 1. One-way ANOVA results comparing parental ssDNA 5mdC measurements to the total gDNA average. Supplementary Table 2. Chromatographic timetable used for iDEMS. Supplementary Table 3. Summary of LC–MS parameters. Supplementary Table 4. Summary of compound-dependent parameters used in iDEMS. (*) Heavy labelled nucleotides feeding cell with (^13^C,d_3_-methyl)-l-methionine; (h) Isotope-labelled internal standard used for quantification.

## Data Availability

All sequencing data generated in this study has been deposited at GEO accession GSE193681. Mass spectrometry data have been deposited in MassIVE as part of the ProteomeXchange Consortium, with the primary accession code MSV000090568. Source data for all relevant panels have been provided. Open source data files (.mzML) converted using msConvert (Proteowizard)^52^ are also available. All other data supporting the findings of this study are available from the corresponding authors on reasonable request. Source data are provided with this paper.

## Source data

Source Data Fig. 1 Statistical source data.
Source Data Fig. 2 Statistical source data.
Source Data Fig. 3 Statistical source data.
Source Data Fig. 4 Statistical source data.
Source Data Fig. 5 Statistical source data.
Source Data Fig. 6 Statistical source data.
Source Data Extended Data Fig. 1 Statistical source data.
Source Data Extended Data Fig. 2 Statistical source data.
Source Data Extended Data Fig. 3 Statistical source data.
Source Data Extended Data Fig. 4 Statistical source data.
Source Data Extended Data Fig. 5 Statistical source data.
Source Data Extended Data Fig. 6 Statistical source data.
