# Supplementary material for: Quantifying propagation of DNA methylation and hydroxymethylation with iDEMS
Source: Nat Cell Biol. 2023 Jan 12;25(1):183–93. doi: 10.1038/s41556-022-01048-x (PMC9859752; doi:10.1038/s41556-022-01048-x)
Supplement: Supplementary file 1 — Supplementary Figs. 1 and 2. [file 41556_2022_1048_MOESM1_ESM.pdf]

nature cell biology

Technical Report

https://doi.org/10.1038/s41556-022-01048-x

# Quantifying propagation of DNA methylation and hydroxymethylation with iDEMS

In the format provided by the authors and unedited

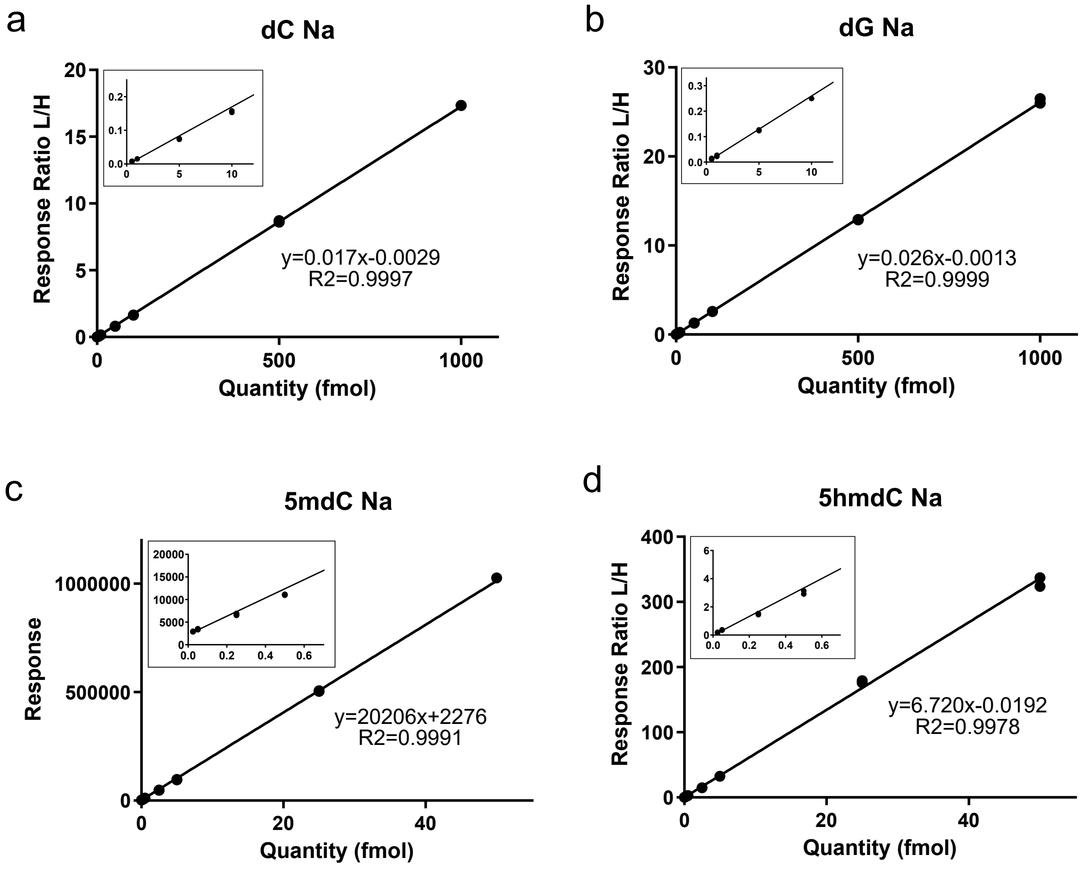


Supplementary Figure 1. Standard curves for the LC-MS/MS nucleoside quantification method for a) dC, b) dG, c) 5mdC and d) 5hmdC. The response for the different quantities of standards in femtomoles is represented as calculated by the mass spectrometry analysis. L/H: “Light” standards/”Heavy” isotope-labelled internal standards.

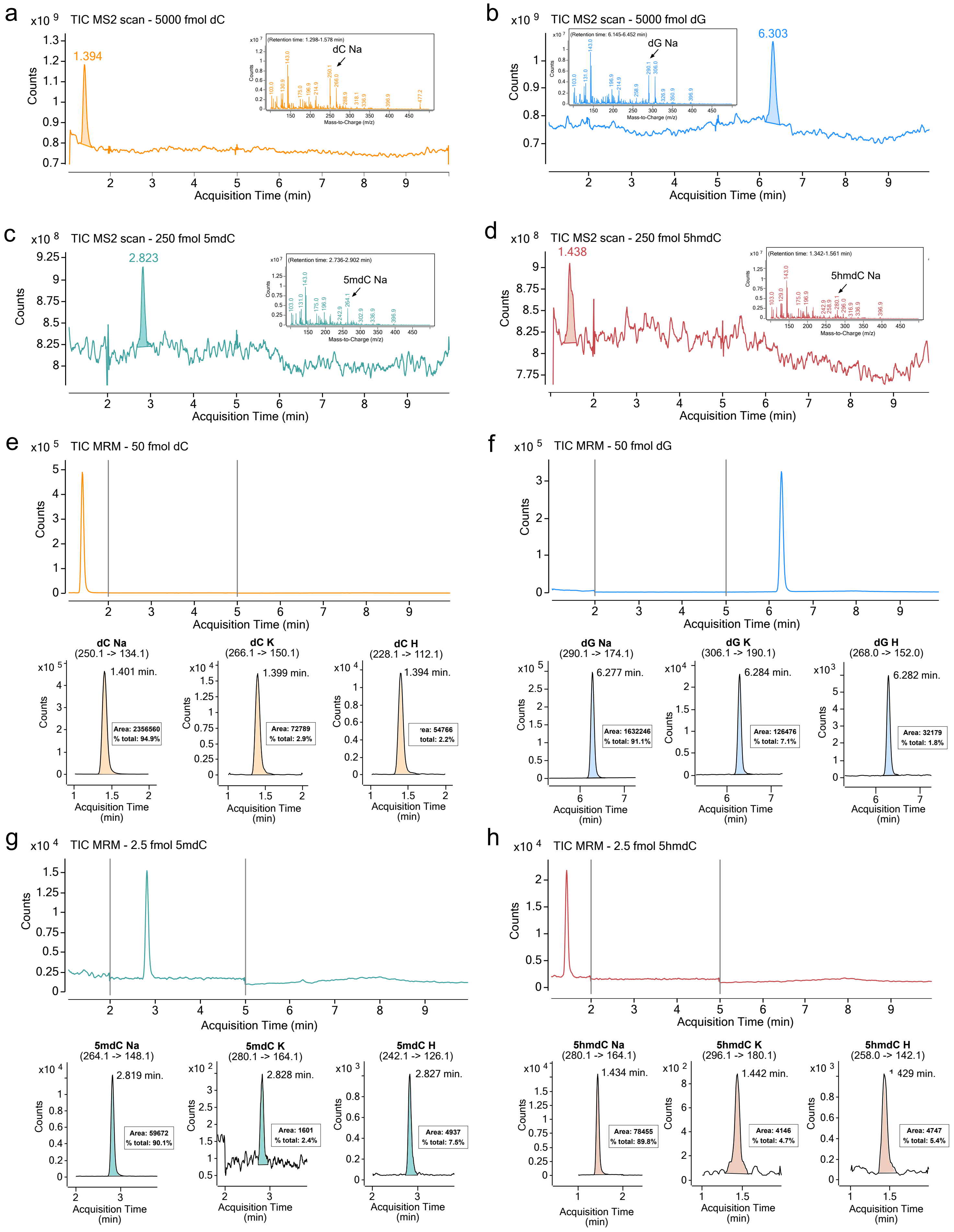

Supplementary Figure 2. Mass spectrometry assessment of nucleoside standards and the generated adducts. Total ionchromatograms (TIC) and inserted the MS2 scan from 100 to 500 m/z for a) dC, b) dG, c) 5mdC and d) 5hmdC. TIC for the standards in MRM mode and the main adducts detected are shown below for e) dC f) dG g) 5mdCh) 5hmdC.
