# Supplementary material for: Quantifying propagation of DNA methylation and hydroxymethylation with iDEMS
Source: Nat Cell Biol. 2023 Jan 12;25(1):183–93. doi: 10.1038/s41556-022-01048-x (PMC9859752; doi:10.1038/s41556-022-01048-x)
Supplement: Supplementary file 2 — Reporting Summary [file 41556_2022_1048_MOESM2_ESM.pdf]

nature portfolio

Corresponding author(s): Anja Groth, Petra Hajkova

Last updated by author(s): Oct 24, 2022

Reporting Summary

Nature Portfolio wishes to improve the reproducibility of the work that we publish. This form provides structure for consistency and transparency in reporting. For further information on Nature Portfolio policies, see our Editorial Policies and the Editorial Policy Checklist.

Statistics

For all statistical analyses, confirm that the following items are present in the figure legend, table legend, main text, or Methods section.

n/a	Confirmed	
☐	☒	The exact sample size (*n*) for each experimental group/condition, given as a discrete number and unit of measurement
☐	☒	A statement on whether measurements were taken from distinct samples or whether the same sample was measured repeatedly
☐	☒	The statistical test(s) used AND whether they are one- or two-sided<br>*Only common tests should be described solely by name; describe more complex techniques in the Methods section.*
☒	☐	A description of all covariates tested
☐	☒	A description of any assumptions or corrections, such as tests of normality and adjustment for multiple comparisons
☐	☒	A full description of the statistical parameters including central tendency (e.g. means) or other basic estimates (e.g. regression coefficient) AND variation (e.g. standard deviation) or associated estimates of uncertainty (e.g. confidence intervals)
☐	☒	For null hypothesis testing, the test statistic (e.g. *F*, *t*, *r*) with confidence intervals, effect sizes, degrees of freedom and *P* value noted<br>*Give P values as exact values whenever suitable.*
☒	☐	For Bayesian analysis, information on the choice of priors and Markov chain Monte Carlo settings
☒	☐	For hierarchical and complex designs, identification of the appropriate level for tests and full reporting of outcomes
☒	☐	Estimates of effect sizes (e.g. Cohen's *d*, Pearson's *r*), indicating how they were calculated

Our web collection on statistics for biologists contains articles on many of the points above.

Software and code

Policy information about availability of computer code

Data collection	EM-seq libraries were sequenced 75 bp paired-end on an Illumina NextSeq 500. Fastq files were generated using bcl2fastq v2.19.1. FastQC v0.11.7<br>(http://www.bioinformatics.babraham.ac.uk/projects/fastqc) was used for QC metrics, and multiqc v1.7 for reporting. Reads were trimmed with trim_galore 0.6.4<br>(https://github.com/FelixKrueger/TrimGalore), mapped to the GRCm38/mm10 mouse reference genome and control genomes with bowtie2 using Bismark v0.22.1 (bismark -N 1 -L 20 -X 1000), and deduplicated with deduplicate_bismark.<br>Mass spectrometry data was measured using Agilent MassHunter acquisition software for LC/MS systems.
Data analysis	Analysis and figure generation were done in HOMER v4.11, GREAT v4.0.4, Seqmonk, v1.47.1; RStudio, v1.3.1093; GraphPad Prism, v9.2.0; FlowJo, v10.8; and Agilent MassHunter Qualitative and Quantitative Analysis Softwares, v10.0.

For manuscripts utilizing custom algorithms or software that are central to the research but not yet described in published literature, software must be made available to editors and reviewers. We strongly encourage code deposition in a community repository (e.g. GitHub). See the Nature Portfolio guidelines for submitting code & software for further information.

Data

Policy information about availability of data

All manuscripts must include a data availability statement. This statement should provide the following information, where applicable:

- Accession codes, unique identifiers, or web links for publicly available datasets
- A description of any restrictions on data availability
- For clinical datasets or third party data, please ensure that the statement adheres to our policy

All sequencing data generated in this study has been deposited at GEO accession GSE193681. Mass spectrometry data have been deposited in MassIVE as part of the ProteomeXchange Consortium, with the primary accession code MSV000090568. Source data for all relevant panels have been provided. Open source data files (.mzML) converted using msConvert (Proteowizard) (Chambers, M. C. et al. A cross-platform toolkit for mass spectrometry and proteomics. Nat Biotechnol 30, 918–920 (2012).) are also available. All other data supporting the findings of this study are available from the corresponding authors on reasonable request.

Field-specific reporting

Please select the one below that is the best fit for your research. If you are not sure, read the appropriate sections before making your selection.

☒ Life sciences ☐ Behavioural & social sciences ☐ Ecological, evolutionary & environmental sciences

For a reference copy of the document with all sections, see nature.com/documents/nr-reporting-summary-flat.pdf

Life sciences study design

All studies must disclose on these points even when the disclosure is negative.

Sample size	All experiments were run in biological triplicate, except for the 12h iDEMS timepoint where four biological replicates were used (Extended Data Figures 2a-b, Extended Data Figures 5b-c, h) and initial iDEMS optimization where technical duplicates were used (Figure 1c). No statistical method was used to predetermine sample size. Sequencing experiments were designed to meet or exceed ENCODE standards (Consortium, E. P. An integrated encyclopedia of DNA elements in the human genome. Nature 489, 57–74 (2012)). For mass spectrometry experiments, our sample size ($n = 3$ for all experiments used in statistical comparisons) was sufficiently powered to detect significance at $p < 0.05$.
Data exclusions	No samples were excluded from analysis.
Replication	All mass spectrometry measurements were run in technical duplicate, with the average from these measurements reported for each sample. All experiments were repeated in at least three independent experiments. All results successfully replicated across independent experiments. Whenever possible, biological replicates were generated from the same lots of reagents for cell culture and sample preparation to minimize technical variation between samples.
Randomization	During timecourse sample preparation, dishes were only labelled from their time of collection to ensure equal treatment of all samples prior to collection and random allocation of dishes into each timepoint. Following sample collection samples were placed in groups based on treatment: timepoint, DNA strand (stranded data), cell population (EM-seq data), or immunoprecipitated histone PTM (ChIP-iDEMS data).
Blinding	Samples were not blinded because no manual quantifications were performed.

Reporting for specific materials, systems and methods

We require information from authors about some types of materials, experimental systems and methods used in many studies. Here, indicate whether each material, system or method listed is relevant to your study. If you are not sure if a list item applies to your research, read the appropriate section before selecting a response.

Materials & experimental systems

n/a	Involved in the study	
☐	☒	Antibodies
☐	☒	Eukaryotic cell lines
☒	☐	Palaeontology and archaeology
☒	☐	Animals and other organisms
☒	☐	Human research participants
☒	☐	Clinical data
☒	☐	Dual use research of concern

Methods

n/a	Involved in the study	
☒	☐	ChIP-seq
☐	☒	Flow cytometry
☒	☐	MRI-based neuroimaging

Antibodies

Antibodies used

H4K20me2: Diagenode, C15200205, 5 µg/ IP
H4K20me0: Abcam, ab227804, 10 µg/ IP
H4K5ac: Abcam, ab51997, 10 µg/ IP
H3K36me3: Abcam, ab9050, 5 µg/ IP
H3K9me3: Abcam, ab176916, 6.4 µg/ IP
anti-rabbit IgG Dynabeads: Invitrogen, 11203D: 150 µL/ IP
anti-mouse IgG Dynabeads: Invitrogen, 11202D: 150 µL/ IP

Validation

All antibodies used are commercially available and have been validated by the manufacturers for ChIP. H4K20me0, H4K5ac, and H3K9me3 antibodies have reactivity in mouse validated by the respective manufacturers; H4K20me2 and H3K36me3 antibodies have reactivity in mouse as previously reported by the Groth lab (Petryk, N. et al. MCM2 promotes symmetric inheritance of modified histones during DNA replication. Science 361, 1389–1392 (2018)).

Eukaryotic cell lines

Policy information about cell lines

Cell line source(s)

Mouse ES cell lines used in this study are E14 background and were gifted by Kristian Helin (commercial source: Mutant Mouse Regional Resource Center at UC Davis) and Joshua Brickman (derived originally by Jan Ure while at the University of Edinburgh). NIH3T3 cells used in this study were gifted by Berthe Marie Willumsen (derived originally by the Doug Lowy lab). Drosophila S2 cells used in this study were obtained from the Drosophila Genomics Resource Center.

Authentication

Cell lines were authenticated based on morphological criteria.

Mycoplasma contamination

All cell lines were tested and found to be negative for mycoplasma.

Commonly misidentified lines
(See ICLAC register)

No commonly misidentified cell lines were used.

Flow Cytometry

Plots

Confirm that:

☒ The axis labels state the marker and fluorochrome used (e.g. CD4-FITC).

☒ The axis scales are clearly visible. Include numbers along axes only for bottom left plot of group (a 'group' is an analysis of identical markers).

☒ All plots are contour plots with outliers or pseudocolor plots.

☒ A numerical value for number of cells or percentage (with statistics) is provided.

Methodology

Sample preparation

To sort G1 and G2M cell populations, mESCs were grown to 70-80% confluence on a coated 15 cm dish and labelled with EdU at a final concentration of 20 µM for 10 minutes. Immediately after labelling, cells were harvested with trypsin. After fixation with ice-cold 70% ethanol, cells were incubated at 4 °C for a minimum of 1 hour. Cells were then spun down at 500 x g for 5 minutes at room temperature and permeabilized in 1X PBS with 1% FCS and 0.25% Triton-X 100 for 10 minutes at room temperature. Cells were spun down at 500 x g for 5 minutes at room temperature and resuspended in 1X PBS with 1% FCS for counting. 5 aliquots of 5 x 106 cells and 3 aliquots of 1 x 106 cells (for single color controls) were transferred to new tubes and spun down at 500 x g for 5 minutes at room temperature. The appropriate tubes were resuspended in Click-IT reaction mix with the following conditions: 1X Click-IT buffer (Click-iT EdU Alexa Fluor 488 Imaging Kit, Thermo Fisher, C10337), 2 mM CuSO4 (from Click-iT kit), 10 mM sodium ascorbate (from Click-iT kit), and Alexa Fluor azide (1:1000 dilution, prepared as directed in Click-iT EdU Alexa Fluor Imaging Kit). Cells were incubated for 30 minutes at room temperature in the dark, then spun down and the appropriate tubes were resuspended in 1X PBS with propidium iodide (10 µg/ml final concentration) and RNAse A (20 µg/ml final concentration). Cells were incubated overnight at 4 °C in the dark before washing and sorting on a BD Aria III flow cytometer. Flow cytometry profiles were analyzed by FlowJo 10.8 software.

For confirmation of cell synchronization and release, mESCs were synchronized and released as described above. Following fixation with ice-cold 70% ethanol, cells were permeabilized and labelled with propidium iodide as described above before analyzing on a LSR Fortessa X20 flow cytometer.

Instrument

BD LSR Fortessa X20, BD FACS Aria III

Software

Data was analyzed with FlowJo, v10.8.

Cell population abundance

Post-sort purity of G1 cells and G2/M cell populations was assessed by re-running a small aliquot of sorted cells immediately after sorting. 400,000-1,000,000 cells were collected for each population from each sort.

Gating strategy

The gating strategy is provided in Figure 4a.

☒ Tick this box to confirm that a figure exemplifying the gating strategy is provided in the Supplementary Information.
